# Impact of arterial catheter location on the accuracy of cardiac output provided by an endotracheal bioimpedance device

**DOI:** 10.1186/cc10828

**Published:** 2012-03-20

**Authors:** F Gennart, S Beckers, C Verborgh, A De baerdemaeker, J Poelaert

**Affiliations:** 1 UZ Brussels, Belgium

## Introduction

With respect to a radial arterial pressure measurement, a more central achievement of this pressure should improve the reliability of endotracheal bioimpedance cardiac output (CO) monitoring. We therefore compared prospectively the impact on accuracy of this device, in comparison with thermodilution (TD) CO.

## Methods

Fourteen patients undergoing cardiac surgery with cardiac output monitoring by TD have been enrolled. A specially designed endotracheal tube (ECOM; ConMed) was placed in conjunction with a catheter located either in the brachial (18 G) or in the radial (20 G) artery in each group of seven patients. Six individual measurements have been carried out in each patient at fixed period, resulting in a total of 42 measurements for each subset. The mean CO by TD was compared with CO by ECOM for each operative period and assessed for agreement by linear regression, Bland-Altmann analysis and percentage error methods. The measurement error should not exceed 30% to be considered as valid, according to Critchley and colleagues.

## Results

**Figure 1:**
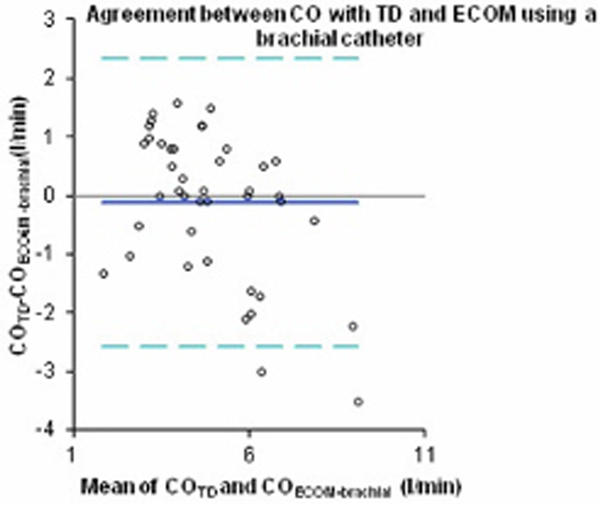


**Figure 2:**
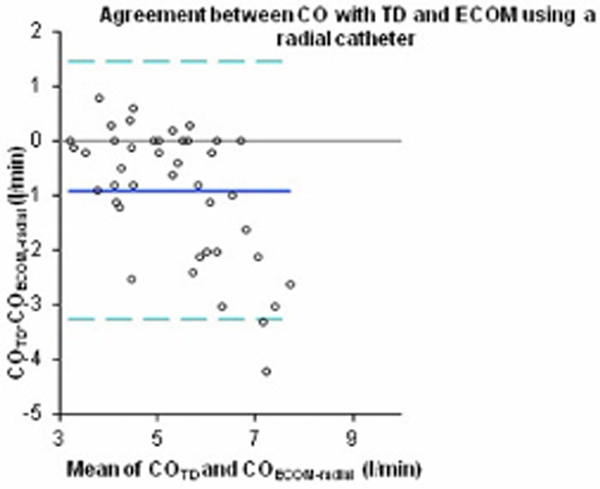


Mean patient age was 71 years (56 to 89) (13 male, one female). R^2 ^values of 0.47 (*P *< 0.01) and 0.63 (*P *< 0.01) in the linear regressions and errors of 41% and 50% were found for the radial and brachial catheter data, respectively. See Figures 1 and 2.

## Conclusion

Accuracy was considerably improved using a brachial artery catheter. Nevertheless, measurement errors between TD and ECOM using either a radial or brachial catheter both exceed 30%. Based on these results and under the current technical conditions, ECOM should not replace TD in CO monitoring for patients undergoing cardiac surgery.
